# Donor Age-Related Biological Properties of Human Dental Pulp Stem Cells Change in Nanostructured Scaffolds

**DOI:** 10.1371/journal.pone.0049146

**Published:** 2012-11-28

**Authors:** Eriberto Bressan, Letizia Ferroni, Chiara Gardin, Paolo Pinton, Edoardo Stellini, Daniele Botticelli, Stefano Sivolella, Barbara Zavan

**Affiliations:** 1 Department of Neurosciences, University of Padua, Padua, Italy; 2 Department of Experimental and Diagnostic Medicine, Section of General Pathology, Interdisciplinary Center for the Study of Inflammation (ICSI) and LTTA Center, University of Ferrara, Ferrara, Italy; 3 Department of Biomedical Sciences, University of Padua, Padua, Italy; 4 ARDEC, Ariminum Odontologica, Rimini, Italy; Robert Wood Johnson Medical School, United States of America

## Abstract

The aim of the present work is to study how biological properties, such as proliferation and commitment ability, of human adult dental pulp stem cells (DPSCs) relate to the age of the donor. Human dental pulps were extracted from molars of healthy adult subjects aged 16 to >66 years. DPSCs were isolated and cultured in the presence of osteogenic, neurogenic, or vasculogenic differentiation medium. Proliferation ability was evaluated by determining doubling time, and commitment ability was evaluated by gene expression and morphological analyses for tissue-specific markers. The results confirm a well-defined proliferative ability for each donor age group at an early in vitro passage (p2). DPSCs from younger donors (up to 35 years) maintain this ability in long-term cultures (p8). Stem cells of all age donor groups maintain their commitment ability during in vitro culture. In vivo tests on the critical size defect repair process confirmed that DPSCs of all donor ages are a potent tool for bone tissue regeneration when mixed with 3D nanostructured scaffolds.

## Introduction

By definition, the main goal of regenerative medicine is to replace or restore the normal function of cells, tissues, and organs that are damaged by means of cell and biomaterials alone or combined. Cells used for this purpose are obtained from a small biopsy of tissue that is dissociated in culture. The resulting cell population is expanded, seeded onto a matrix, and implanted back into the host [1].

In recent years, stem cell biology has emerged as a foundation of regenerative medicine. In this context, dental research has taken advantage of the potential of these cells for clinical applications for the regeneration of endodontic tissues [2]–[4] with mesenchymal stem cells (MSCs). [5], [6]. Several tissue sources could be used for the recruitment of MSCs, such as bone marrow, adipose tissue and dental pulp. MSCs from dental pulp (DPSCs) display multidifferentiation potential, with the capacity to give rise to at least 3 distinct cell lineages: 1 osteo/odontogenic, 2 adipogenic, and 3 neurogenic [7]. DPSC lines are capable of long-term cultivation without changing their viability, phenotype, or genotype. DPSCs express many stem cells markers in distinct patterns from other adult stem cells. Kerkis et al. [8] report that DPSCs do not express hematopoietic marker CD34 or CD45. In contrast they do express high levels of mesenchymal markers STRO-1, CD29, CD44, CD73, CD90, and CD166. For this reason, DPSCs are related to mesenchymal stem cells isolated from the bone marrow, although they differ in some other markers. Their expression of the pluripotent embryonic stem cell marker Sox-2 confirms the primitive nature of DPSCs. Neural stem cell markers such as nestin and nucleostemin that are expressed in DPSCs may reflect the neural crest origin of the dental pulp [8], [27], [28]. DPSCs have been used for tissue-engineering studies in large animals to assess their potential in pre-clinical applications [9], [10].

With the goal of using DPSCs for human endodontic tissue repair, such as bone regeneration, we started our study by asking whether it is reasonable to apply alveolar bone tissue regeneration methods to aged donor stem cells.

In light of such considerations, we studied the biological properties of human DPSCs in relation to the age of the donor, classified into 6 different groups. Proliferation ability and differentiation potential were analyzed by cell biology, molecular biology, and immunocytochemical analyses. Their in vivo regenerative ability was also examined. The correlations of donor age and in vitro aging with biological properties, such as commitment ability, were then evaluated and discussed.

## Materials and Methods

### Biomaterial

Hydroxyapatite (HA)-based scaffolds were supplied in granules (Maxresorb®, Botiss, Germany). They were placed in a cell suspension (1×10^6^ cells per scaffold) under vacuum conditions for 60 s to facilitate the flow of the cells inside the pores. After 3 hours of incubation at 37°C with 5% CO_2_, the scaffolds were cultured in medium for 2–3 weeks at 37°C with 5% CO_2_. *In vivo*, the control scaffolds were treated identically, but without cells.

### Dental pulp extraction and differentiation

Human dental pulps were extracted from healthy molar teeth, which had been extracted because of mucosal inflammation (impacted teeth with pericoronitis) or for orthodontic reasons from adult subjects aged 16 to >66. The pulps were classified into 6 age groups (12 teeth per group).

Each subject gave informed written consent for the use of their donor of dental pulps. The Ethical Committee of Padua Hospital approved the research protocol. Before extraction, each subject was checked for systemic and oral infections or diseases. Only disease-free subjects were selected for pulp collection. Each subject was pretreated for 1 week with professional dental hygiene. Before extraction, the dental crown was covered with a 0.3% chlorexidin gel (Forhans, New York, NY) for 2 min. After mechanical fracturing, dental pulp was obtained by means of a dentinal excavator or a Gracey curette. The pulp was gently removed and immersed for 1 h at 37°C in a digestive solution: 100 U/ml penicillin, 100 mg/ml streptomycin, 0.6 ml of 500 mg/ml clarithromycin, 3 mg/ml type I collagenase, and 4 mg/ml dispase in 4 ml of 1 M PBS. Once digested, the solution was filtered through 70 mm Falcon strainers (Becton & Dickinson, Franklin Lakes, NJ).

### Cell culture

For each single pulp sample, cells were in vitro-cultured as a monolayer up to p1 in non-differentiative medium. Afterwards, they were seeded in 7 different well plates and cultured in the following differentiation media (7 different conditions for each sample) up to 21 days.

#### Non-differentiative medium

Non-hematopoietic (NH) stem cell expansion medium (Miltenyi Biotec, Bergish Gladbach, Germany).

#### Osteogenic differentiation medium

NH OsteoDiff Medium (Miltenyi Biotec, Bergish Gladbach, Germany).

#### Endothelial differentiation medium

DMEM containing 10% FBS plus 0,1 ng/ml human recombinant ECGF, 10 µg/ml human bFGF (Calbiochem, San Diego, CA) and 100 µg/ml porcine heparin (Seromed; Berlin, Germany).

#### Neuronal differentiation medium

DMEM-F12 3∶1 containing 1% FBS, 2% b27 serum-free supplement, 10 µg/ml NGF, antibiotics.

#### Glial differentiation medium

DMEM-F12 3∶1 containing 1% FBS plus 1% N2 supplement, 4 µM forskolin, and 10 ng/ml heregulin ß.

#### Chondrogenic differentiation medium

NH ChondroDiff Medium (Miltenyi Biotec, Bergish Gladbach, Germany).

#### Adipogenic differentiation medium

NH AdipoDiff Medium (Miltenyi Biotec, Bergish Gladbach, Germany).

### Growth curve and doubling time

Cells were seeded into 6-well plates at an initial density of 5×10^4^. When cells reached confluence, they were detached, counted and re-seeded at a density of 5×10^4^. The PDT of the cells was calculated according to the formula:
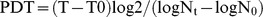
where PDT represents the cell doubling time, t represents the duration of cell culture, and N_0_ and N_t_ represent the cell number after seeding and the cell number after culturing for t hours, respectively [29].

### Statistical analysis

One-way analysis of variance (ANOVA) was used for data analyses. The Leveneís test was used to demonstrate the equal variances of the variables. Repeated-measures ANOVA with a post-hoc analysis using Bonferroni's multiple comparison was performed. T tests were used to determine significant differences (p<0.05). Repeatability was calculated as the standard deviation of the difference between measurements. All testing was performed in SPSS 16.0 software (SPSS Inc., Chicago, Illinois, USA) (license of the University of Padua, Italy).

### Immunocytochemical staining

DPSCs were layered over cover slip, fixed with absolute acetone for 10 min at room temperature and cryopreserved at −20°C until use. The following markers were visualized with immunofluorescence: CD31, osteonectin, S100, nestin, GFAP (monoclonal mouse anti-human; SIGMA), βIII tubulin, and CNPase (monoclonal rabbit anti-human; SIGMA). Briefly, after non-specific antigen sites were saturated with 1/20 serum in 0.05 M maleate TRIZMA (Sigma; pH 7.6) for 20 sec, 1/100 primary monoclonal anti-human Ab (Sigma) was added to the samples. After incubation, immunofluorescence staining was performed with fluorescein (anti-rabbit) rhodamine (anti-mouse) secondary antibody.

### Quantitative analyses of differentiation

The percent of differentiated cells was calculated by counting the positive cells for each marker compared to the total number of cells present on the coverslip.

### Real-Time PCR

Primers and probes were selected for each target gene using Primer3 software. Gene expression was measured using real-time quantitative PCR on a Rotor-Gene 3500 (Corbett Research, Sydney, Australia). PCRs were carried out using the designed primers at 300 nm and SYBR Green I dye (Invitrogen, Carlsbad, CA) (using 2 mm MgCl2) with 40 cycles of 15 s at 95°C and 1 min at 60°C. All cDNA samples were analyzed in duplicate. The cycle threshold (Ct) was determined automatically by the software. The amplification efficiencies of the studied genes was 92 to 110%. For each cDNA sample, the Ct of the reference gene, L30, was subtracted from the Ct of the target sequence to obtain the ΔCt. The expression level was then calculated as 2ΔCt and expressed as the mean ± SD of quadruplicate samples of two separate runs. Experiments were performed with three different cell preparations and repeated at least three times. As control DPSCs, human fibroblasts cultured in the presence of basal culture medium for undifferentiated DPSCs were used. The baseline in the “basal culture medium for undifferentiated DPSC” panel is the expression in fibroblast cell culture. The baseline in the other panels is the expression of the markers in DPSCs cultured in basal culture medium for undifferentiated DPSCs. The *in vivo* experimental results are expressed as fold expression increases of human markers (in samples of stem cells on nanostructured material) compared to rat markers in samples of nanostructured material without stem cells.

The human primer sequences are listed in table 1.

**Table 1:** Primer sequences.

	FOR	REV	Product (bp)
von Willebrand factor (VWF)	ACGTATGGTCTGTGTGGGATC	GACAAGACACTGCTCCTCCA	159
CD31	TCCAGCCAACTTCACCATCC	TGGGAGAGCATTTCACATACGA	171
OSTEONECTIN	TGCATGTGTCTTAGTCTTAGTCACC	GCTAACTTAGTGCTTACAGGAACCA	186
OSTEOCALCIN	GCAGCGAGGTAGTGAAGAGAC	AGCAGAGCGACACCCTA	193
OSTEOPONTIN	TGGAAAGCGAGGAGTTGAATGG	GCTCATTGCTCTCATCATTGGC	192
S100	GACAAGTACAAGCTGAGCAAGAAG	CCACAAGCACCACATACTCCTG	166
NESTIN	TCAGAGGGAAGGAGATAGAGAGTC	AGCCAGAAACCATATGTCAAGAGA	171
GFAP	AGATCCGCACGCAGTATGAG	AGGTCGCAGGTCAAGGA	178
CNPase	AGATGCGGTGGCTAAAGGTC	TCTTAGGCAGCTCTTTGGGA	153
COLL I	TGAGCCAGCAGATCGAGA	ACCAGTCTCCATGTTGCAGA	178
COLL II	CAGCAAGAGCAAGGAGAAGAAAC	GTGGTAGGTGATGTTCTGGGA	163
PPAR gamma	CAGGAGATCACAGAGTATGCCAA	TCCCTTGTCATGAAGCCTTGG	149
ADIPONECTIN	GATGAGAGTCCTGGGTGTGAG	CTGGGTAGATATGGGATTCAAGAGA	148
GLUT 4	CCTGATCATTGCGGTCGTG	CCGAGACCAAGGTGAAGACTG	163

### *In vivo* model

In vivo rat calvarial defect models of bone regeneration using HA scaffolds were prepared for calvarial implantation in 24 8-week-old female nude rats (Wistar-NIH-FOXN1, Charles River). Two defects were prepared for each rat: HA scaffolds alone (control) and HA scaffolds+DPSCs (from the younger donor group (16–25) or from the senior group (over 66) at a density of 1×10^7^ cells/ml). All operations were performed under general anesthesia achieved with intraperitoneal injection of ketamine hydrochloride (Ketaras, Yuhan Corp. Korea, 40 mg/kg) mixed with xylazine (Rumpens Bayer Korea Ltd., Korea, 10 mg/kg). After disinfecting the calvarial skin with 10% Betadine (Potadines, Sam-Il Pharm., Korea) and subcutaneous injection of 2% lidocaine containing 1∶100,000 epinephrine (Lidocaine HCL Injs. Yuhan Corp., Korea) into the calvarial bone, an incision was made along the sagittal suture. The periosteum was elevated, and a calvarial bone defect 5 mm in diameter was created with a trephine burr without perforating the dura. The area of the defect was either left with HA scaffolds alone (control group) or filled with HA+DPSCs (experimental group). The animals were sacrificed 3 weeks after surgery under formalin perfusion. The calvarial bone was removed from the skull and decalcified. The decalcified bone tissues were fixed in 10% formalin overnight and embedded in paraffin after dehydration in 70% ethanol. For histochemical analysis, the paraffin sections were fixed for 10 min with xylene and stained with H&E (Sigma) and Masson's trichrome (MT) to detect cells and bone structures. All the animals were treated and handled in accordance with the “Recommendations for Handling Laboratory Animals for Biomedical Research” compiled by the Committee on the Safe and Ethical Handling Regulation for Laboratory Experiments at the University of Padua. The animals were housed separately in thermostat-controlled cages (22°C) with a 12-h day/night cycle and food available ad libitum and were unrestrained.

### Histological staining

The cellularized scaffolds were fixed in 4% paraformaldehyde phosphate-buffered saline (PBS, Seroderm, Berlin, Germany) for 72 h and then dehydrated in a graded series of ethanol and acetone steps. Serial 7-mm sections were cut perpendicular to the osseous defects and surrounding bone (Reichert-Jung 2050, Nussloch, Germany). The bone sections were stained with Van Gieson stain and observed under a light microscope.

### Ethics Statement

The Ethical Committee of Padua Hospital approved the research protocol. Each subject gave informed written consent for use of their of dental pulp. The Ethics Committee of Padua Hospital approved the research protocol. We obtain informed consent from the next of kin, caretakers, or guardians on the behalf of the minors/children participants (under 18 of age)

The animal protocol was approved by the Institutional Animal Care Committee of Padua University and conducted in compliance with U.S. National Research Council guidelines for the ethical treatment of animals.

## Results

### Stemness of DPSCs

**Figure 1:**
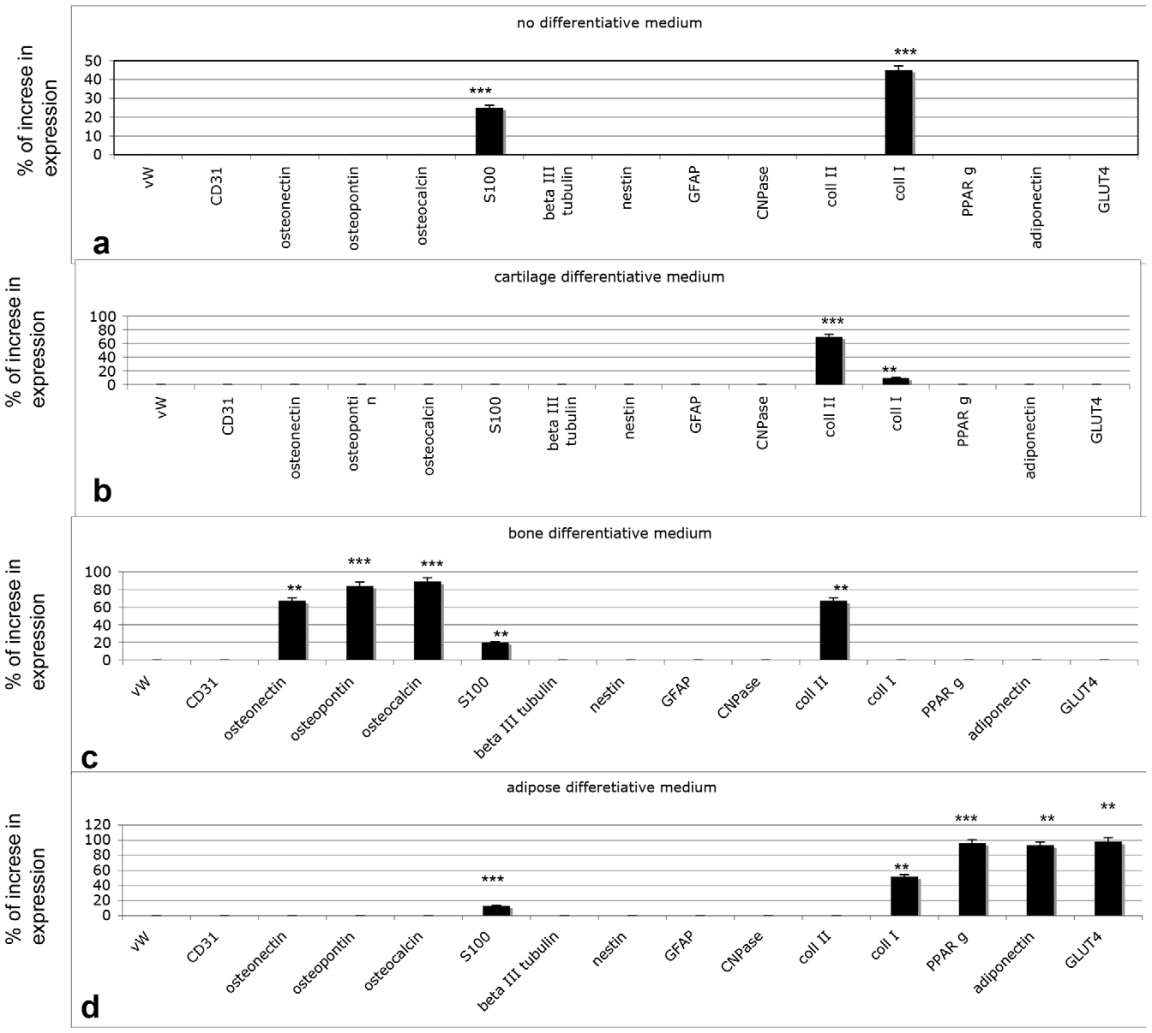
Gene expression according to real-time PCR on undifferentiated DPSCs in basal culture medium (A) and in media that induce differentiation: chondrogenic (B), osteogenic (C) or adipogenic (D). One-way analysis of variance (ANOVA) was used for data analyses. The Leveneís test was used to demonstrate the equal variances of the variables. Repeated-measures ANOVA with a post-hoc analysis using Bonferroni's multiple comparison. T tests were used to determine significant differences (p<0.05). * p<0,05; * * p<0,01; * * * p<0,001. Repeatability was calculated as the standard deviation of the difference between measurements.

The isolation of DPSCs from human dental pulp and their *in vitro* differentiation capacity into various mesenchymal tissues were first established by using standard protocols. Under these conditions (using specific differentiation media), the classical adipogenic, osteogenic, and chondrogenic media were highly efficient in causing specific differentiation into the expected cell lineages, as confirmed by molecular hallmarks (Fig. 1). Moreover, we analyzed the commitment to these lineages versus the expression of endothelial, neuronal and glial like features.

First, to confirm that cells isolated from dental pulp with our protocols were dental pulp stem cells (DPSCs), we cultured the cells in the presence of basal culture medium for undifferentiated DPSCs (Fig. 1a), and we performed gene expression analyses to detect their phenotypes. After 2 days of culture in basal culture medium for undifferentiated DPSCs, real-time PCR was performed. The genes selected for this screening were the following:

Endothelial commitment: von Willebrand factor (vWF) and CD31. As reported in Fig. 1a, no expression for these markers was detectable.

Bone commitment: osteopontin, osteonectin, and osteocalcin. No expression of these markers was detectable (Fig. 1).

Neuronal commitment: S100, βIII tubulin, and nestin. Low expression of S100 was detectable, whereas no expression of other markers was found.

Glial commitment: GFAP and CNPase. Neither of these was detectable.

Adipogenic commitment: adiponectin, GLUT4, PPARγ. No expression was found.

Fibroblastic commitment: collagen type I was detected at 45% of the expression level of the control.

Twin cultures were then performed for 21 days in different media: chondrogenic (Fig. 1b), osteogenic (Fig. 1 c), and adipogenic (Fig. 1 d). The gene expression patterns of the cultures confirmed the correct commitment. Indeed, in chondrogenic medium, only collagen type II was detectable, and no endothelial or neuronal markers were present. In osteogenic medium, collagen type I, osteopontin, osteonectin, and osteocalcin were abundant, whereas no traces of collagen type II or adipogenic genes were observable. In adipogenic medium, no neuronal, endothelial, bone or cartilage markers were detected. PPARγ, adiponectin and GLUT4 (specific for adipogenesis) were observed (Fig. 1d).

### Proliferative activity

Population doubling time (PDT) is used to evaluate the ability of the cell to duplicate in number and is therefore a direct marker of the proliferative ability of the cell. In this experiment, we analyzed the PDT of DPSCs cultured in non-differentiative medium. PDT was evaluated at 3 different in vitro passages (p) of the cultures:

- p2: early passage
- p5: medium-term culture
- p8: long-term culture

**Figure 2:**
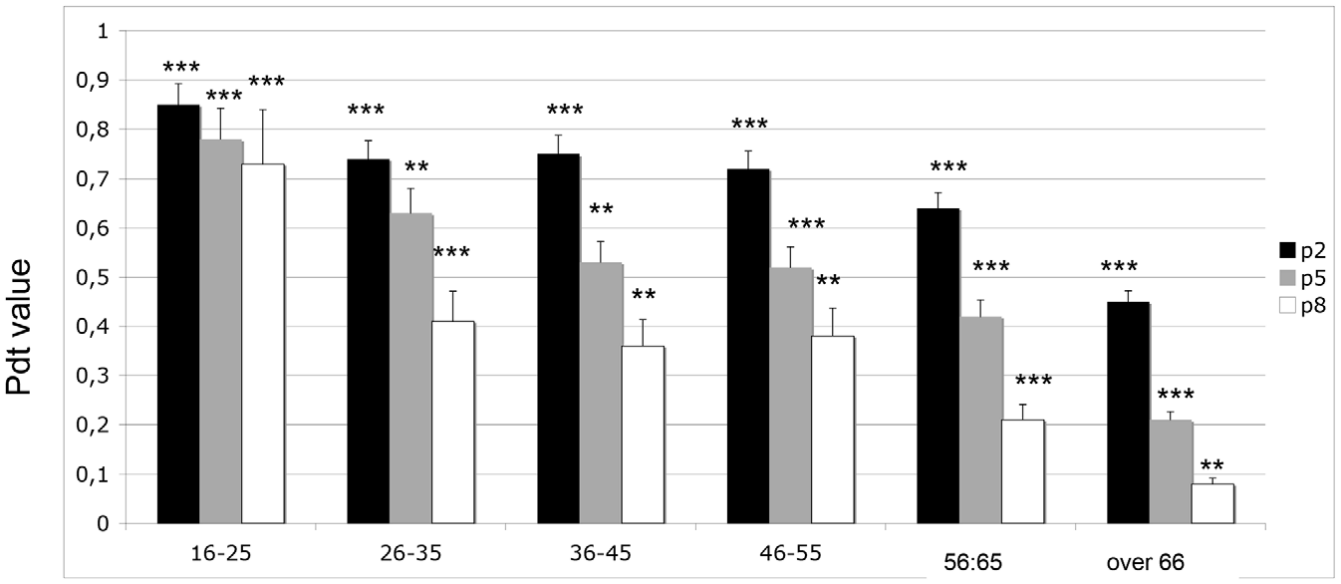
Population doubling time (PDT) of DPSCs cultured in the presence of basal culture medium for undifferentiated DPSCs. The graph shows in vitro passage (starting from p2 for young cells (black bar) to p8 (white bar)) and the age of the donor (from 16 to over 67). T tests were used to determine significant differences (p<0.05). * p<0,05; * * p<0,01; * * * p<0,001.

As reported in fig. 2, we observed well-defined cell growth for each passage in each age class. For the aged group (up to 67 years), proliferative ability decreased in time and during in vitro aging. This property was not evident for stem cells derived from young donors (up to 25 years), as a high PDT value was maintained in all cell passages. These data confirm the good proliferative ability of stem cells. After age 25, this ability decreased in proportion to the in vitro passage. Interestingly, up to age 56, a high level of proliferation was detectable at p2.

### Qualitative analyses of lineage commitment

**Figure 3:**
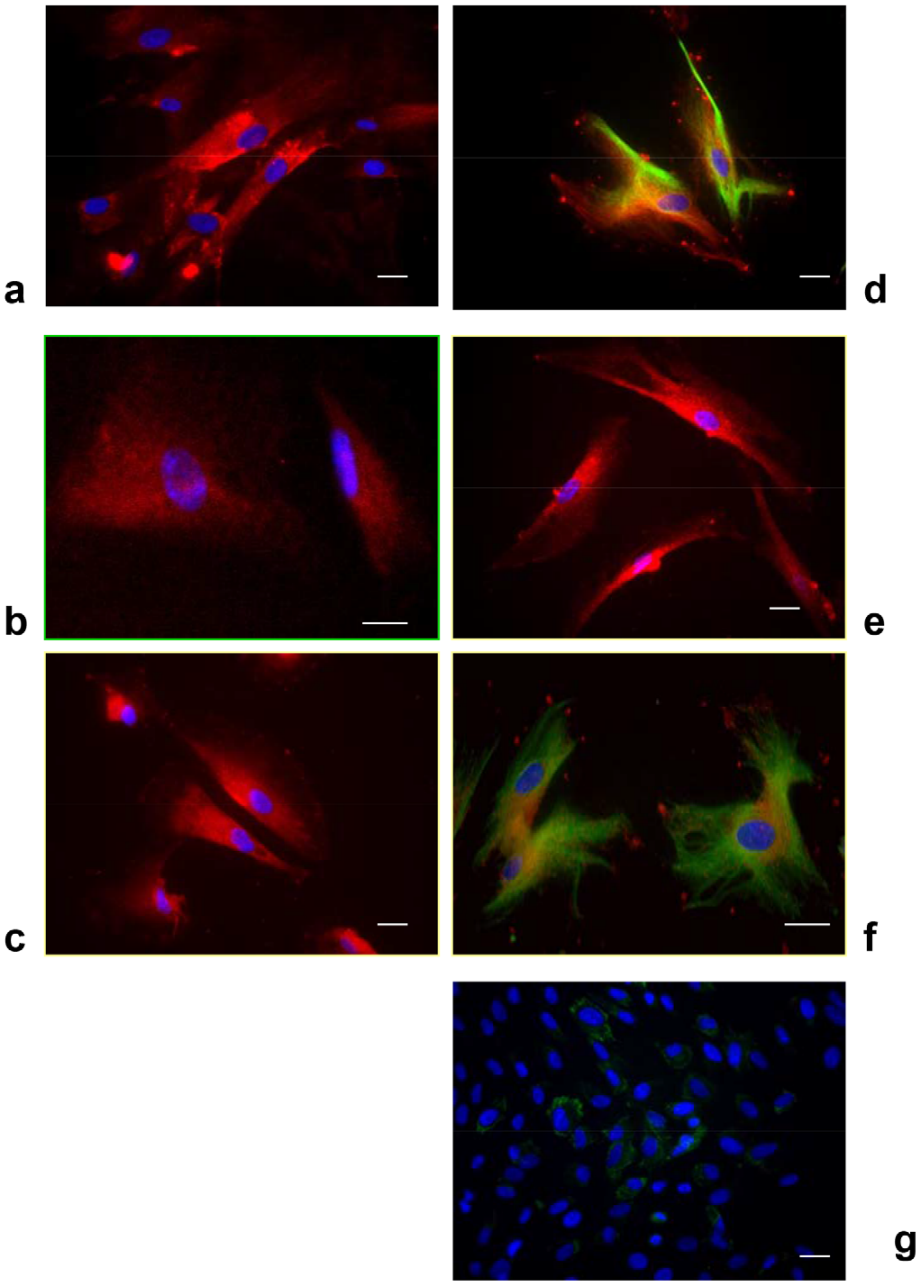
Immunofluorescence analyses of DPSCs committed to several cell lineages. (A) CD31 for endothelial cells (red cells). (B) Osteonectin for osteogenic commitment. (C) S100 (red cells, for neurogenic commitment), (D) nestin (red fibers) and βIII tubulin (green), (E) GFAP (red staining), (F) CNPase (green) and nestin (red) (40×) for glial-like commitment. (G) Negative control: DPSCs cultured in non-differentiative medium and stained with primary Ab against GFAP (no red-positive cell are detectable) and primary Ab against CNPase (no green-positive cells are detectable). Bar: 30 µm.

**Figure 4:**
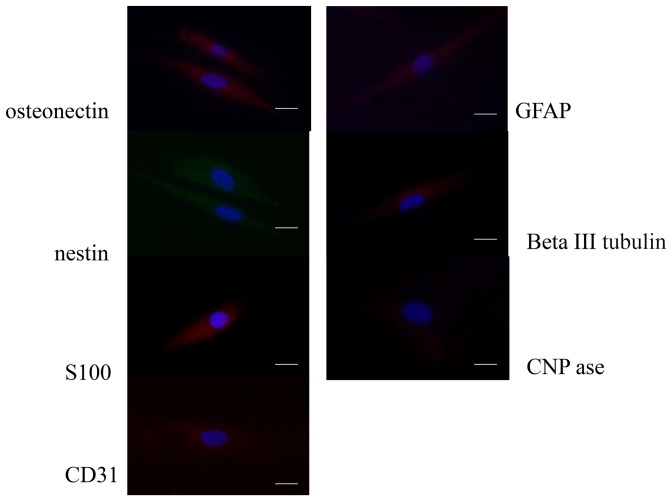
Immunofluorescence analyses of human fibroblasts (used as control). Bar: 30 µm.

**Figure 5:**
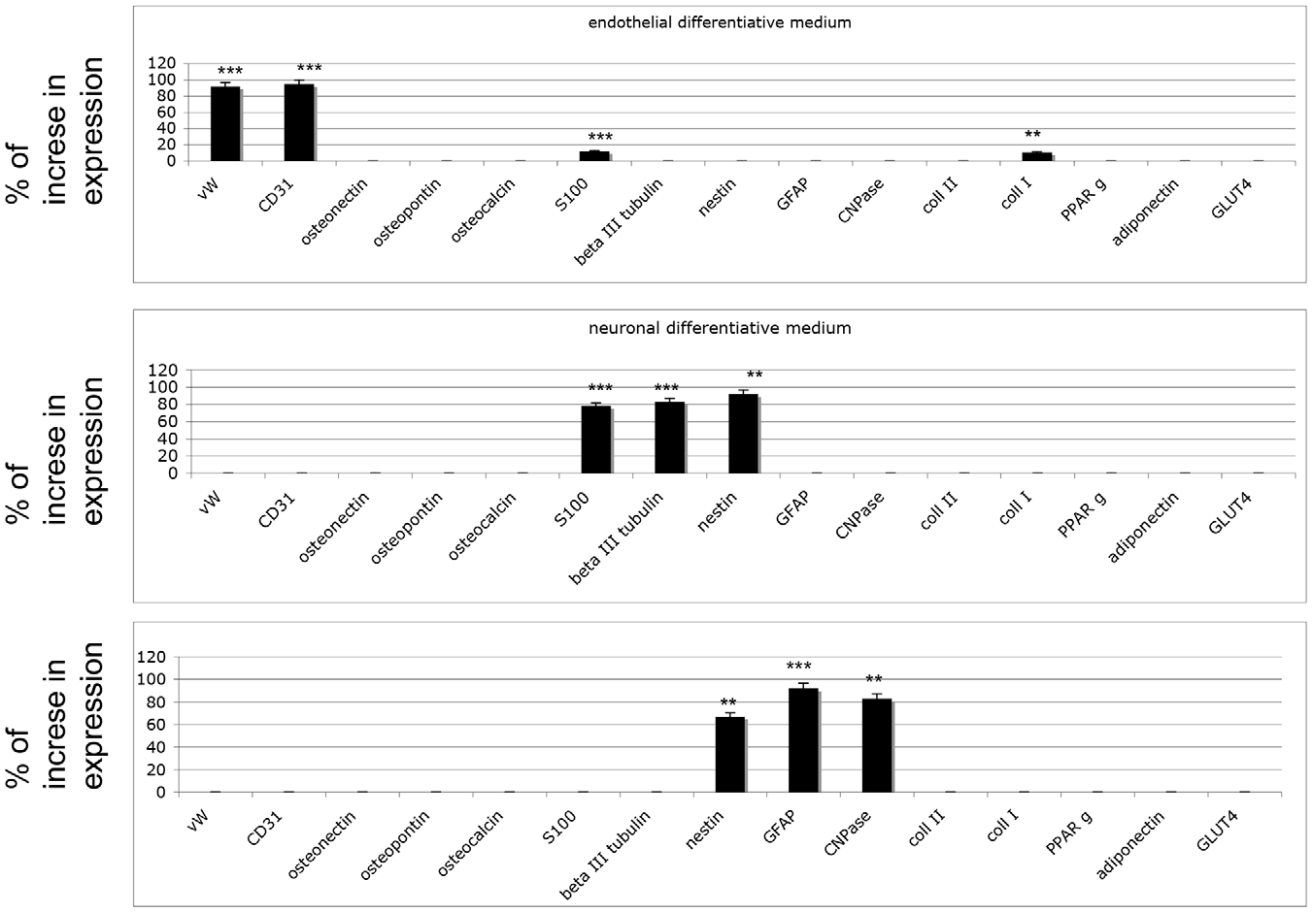
Gene expression by real-time PCR on DPSCs in endothelial, neuronal or glial differentiation medium. One-way analysis of variance was used for data analyses. T tests were used to determine significant differences (p<0.05). * p<0,05; * * p<0,01; * * * p<0,001. Repeatability was calculated as the standard deviation of the difference between measurements.

Qualitative and quantitative analyses of stemness were performed, starting from the data reported in the literature [10]–[16]. The commitment ability of DPSCs was tested by culturing them in the presence of differentiation media. Markers for endothelium, bone, and nervous system were detected by immunostaining (Fig. 3 and Fig. 4 for negative controls) and real-time PCR (Fig. 5). As control, DPSCs cultured in basal culture medium for undifferentiated DPSCs was. To test the specificity of the antibodies used for immunostaining, fibroblast cells were used as control. To analyze the results, we determined whether DPSCs always expressed the correct specific markers in the presence of a given differentiation medium. If DPSCs expressed the expected markers, we referred to them as maintaining that differentiation phenotype, and if they did not, we referred to them as losing this ability.

### Endothelial markers

Specific markers for endothelial commitment, CD31 and vWF, were selected. DPSCs cultured in the presence of endothelial differentiation medium expressed CD31, as indicated by the well-defined red staining (fig. 3 a). Thus, the DPSCs correctly committed to the endothelial phenotype. Corroborating the immunocytochemistry results, the gene expression (Fig.Fig. 5) of CD31 and vWF also indicated endothelial differentiation.

### Bone markers

Osteonectin, a bone-specific protein that binds selectively to both hydroxyapatite and collagen, was selected as a marker of commitment to bone cells. Osteonectin links the bone mineral and collagen phases, perhaps initiating active mineralization in normal skeletal tissue. In the osteogenic medium, the red positive reaction (Fig. 3b) indicated the novel bone phenotype acquired by the cells. This result was supported by the expression of osteopontin, osteocalcin and collagen type I (Fig. 5).

### Neuronal markers

To analyze neuronal commitment capacity, we detected S100 (Fig. 3c), a calcium-binding protein normally present in cells derived from the neural crest (Schwann cells and glial cells), in mesenchymal-derived cells such as chondrocytes and adipocytes, and in dendritic cells. In fig. 3c, it is evident that DPSCs expressed S100, as confirmed by the red fluorescent staining. Nestin, a type VI intermediate filament (IF) protein, is a protein marker for neural stem cells because it is mostly expressed in nerve cells. In our monolayer cell cultures, DPSCs in the presence of neuronal medium expressed nestin (Fig. 3d, red staining), further indicating neuronal commitment. The last protein tested for the neuronal phenotype was βIII tubulin, a protein abundant in the central and peripheral nervous systems, where it is prominently expressed during fetal and post-natal development. All of the DPSCs cultured in neuronal differentiation medium showed a positive expression of this neuronal protein (Fig. 3d, green staining; Fig. 4, negative control). The gene expression detected with real-time PCR (Fig. 5) confirmed the immunocytochemistry results.

### Glial markers

During gliogenesis, nestin is replaced by other IF proteins, such as glial fibrillary acidic protein (GFAP). In our cultures, indeed, no cells expressed GFAP if cultured in neuronal medium (data not shown), whereas in the presence of glial medium, a well-defined cytoskeletal structure positive for GFAP was evident (Fig. 2e, red staining). Another important signal related to glial phenotypes was the co-expression of nestin and CNPase (Fig. 2f). CNPase (2′,3′-cyclic nucleotide 3′-phosphodiesterase) is expressed at high levels by oligodendrocytes in the central nervous system and by Schwann cells in the peripheral nervous system (fig. 2f, green staining). Its co-expression with nestin (Fig. 2f, red staining) confirmed the commitment of DPSCs to the correct phenotype. In Fig. 5, the gene expression levels of these proteins are shown.

### Quantitative analyses

**Figure 6:**
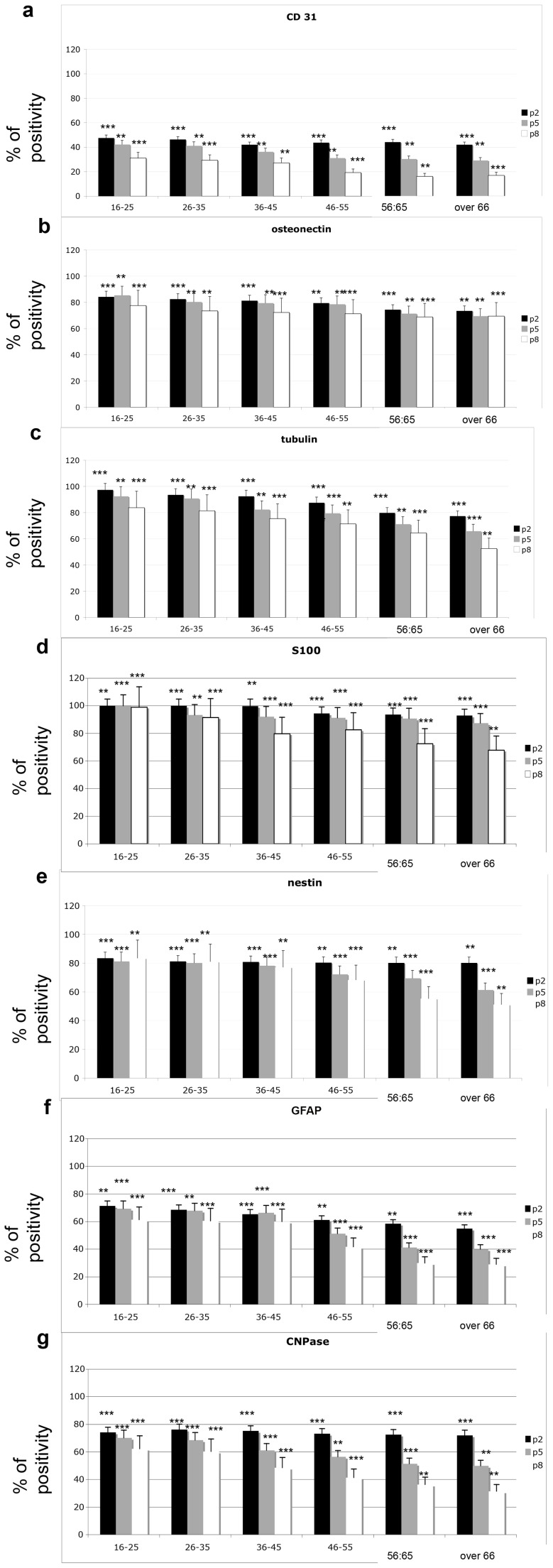
Quantitative analyses of lineage commitment were performed by analyzing the percent of positive cells for each marker. The commitment was detected in all donor age and in vitro passage groups. T tests were used to determine significant differences (p<0.05). * p<0,05; * * p<0,01; * * * p<0,001. Repeatability was calculated as the standard deviation of the difference between measurements.

We analyzed the percent of positive cells for each marker and compared their lineage commitment abilities. These analyses were performed at the 3 most significant in vitro passages of DPSCs (P2, P5, P8) for each age group (Fig. 6) to test if the stemness of DPSCs is related to the *in vitro* (passage) and *in vivo* (age of donor) aging of the cells.

### Endothelial commitment

The commitment into endothelial cells was detected in all groups analyzed. Specifically, young in vitro cultures (namely, p2) showed good commitment (approximately 40% of total cells) into endothelial cells for all age groups analyzed (Fig. 6a, black bar). This behavior was maintained as cells aged: the cells at p5 (gray bar) showed a similar percentage of endothelial commitment as p2 for all age groups. When cells aged in vitro (p8, white bar), they lost this ability in proportion to the age of the donor.

### Bone commitment

A marked in vitro commitment of DPSCs was detectable for each in vitro passage and for each age. Fig. 6b shows that approximately 80% of DPSCs from both p2 and p5 acquired a bone phenotype when they were derived from patient of less of 55 years old. After this age, a strong commitment (approximately 70%) was still evident up to p8.

### Neuronal commitment

Nestin- (Fig. 6c) and S100-positive cells (Fig. 6d) were present at higher levels (100% of the cell population) during p2 (black bars) from donors up to 36 years old. In cells from donors up to 45 years old, Nestin (Fig. 6e) was detectable in a smaller percentage of cell the population (80%), but its expression endured during the *in vitro* aging (from p2 to p8). Cells from senior donors showed a decrease in this marker over time *in vitro*.

GFAP (Fig. 6f) and CNPase (Fig. 6g) were present in approximately 70% of the cells in all p2 cultures for all ages. Starting from 45 years old, their presence dramatically decreased at p5 (gray bar) and at p8 (white bar).

### 3D cultures

**Figure 7:**
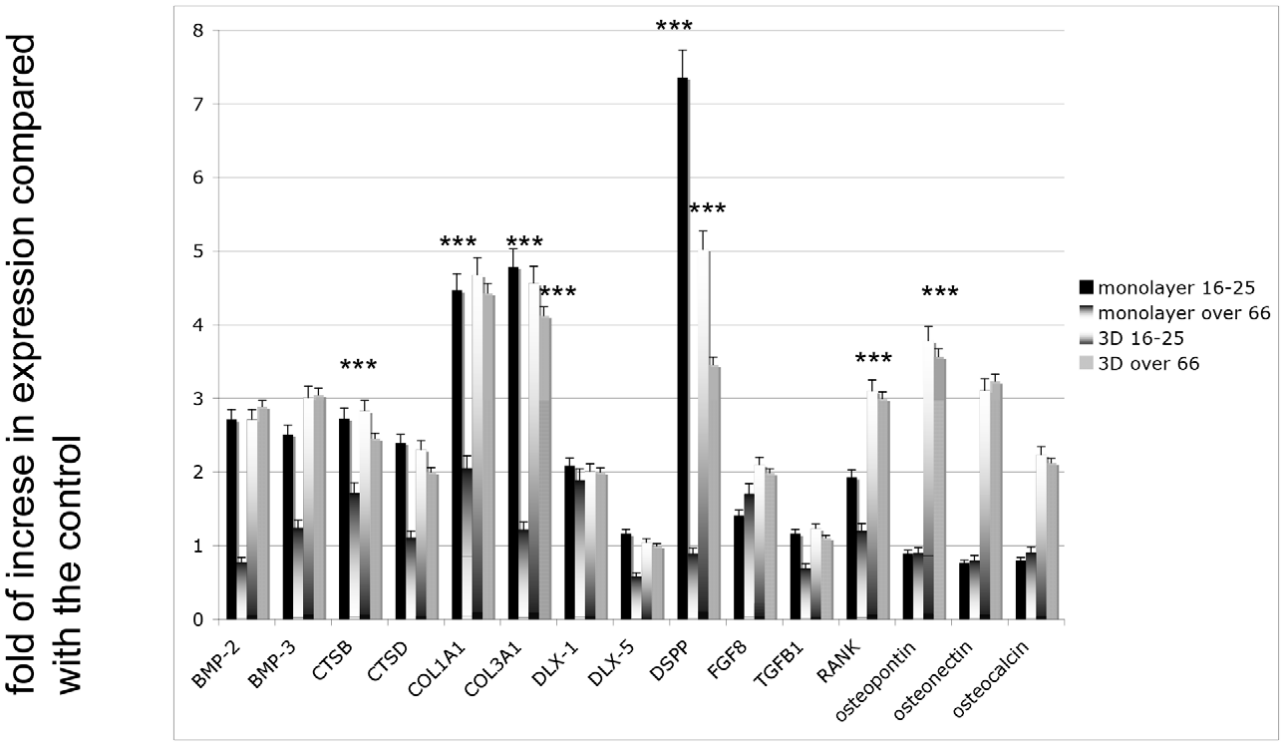
Gene expression by real-time PCR on DPSCs derived from the younger donor group (16–25) and from the senior group (over 66) in monolayers and in nanostructured scaffolds. Markers selected were bone morphogenic protein (BMP) 2, BMP-3, cathepsin B (CTSB), CTSD, collagen type I (Col1A1), collagen type III (Col3A1), Distal-less homeobox (DLX) 1, DLX5, fibroblast growth factor (FGF), transforming growth factor β1 (TGFβ1), receptor activator of nuclear factor kappa-B (RANK), osteopontin, osteonectin, and osteocalcin. The results for each experiment are from quadruplicate experiments. Values are expressed as the mean ± SD. T tests were used to determine significant differences (p<0.05). * p<0,05; * * p<0,01; * * * p<0,001.

Cells of the most representative groups, the 16–25 age group and the senior age group (over 66) at p5, were seeded onto HA granules, and their osteogenic potential was evaluated. The marker expression in monolayer conditions versus 3D conditions were then compared. As shown in Fig. 7, the presence of HA considerably improved the osteogenic population: osteopontin, osteonectin, osteocalcin, collagen type I, collagen type III, cathepsin B (CTSB), cathepsin D CTSD, distal-less homeobox 1 (DLX1), DLX5, dental sialoprotein (DSPP), fibroblast growth factor 8 (FGF8), transforming growth factor β1 (TGF-β1), and RANK were more strongly expressed when cells where mixed with HA than in monolayer conditions from both donor age groups.

### *In vivo* findings

**Figure 8:**
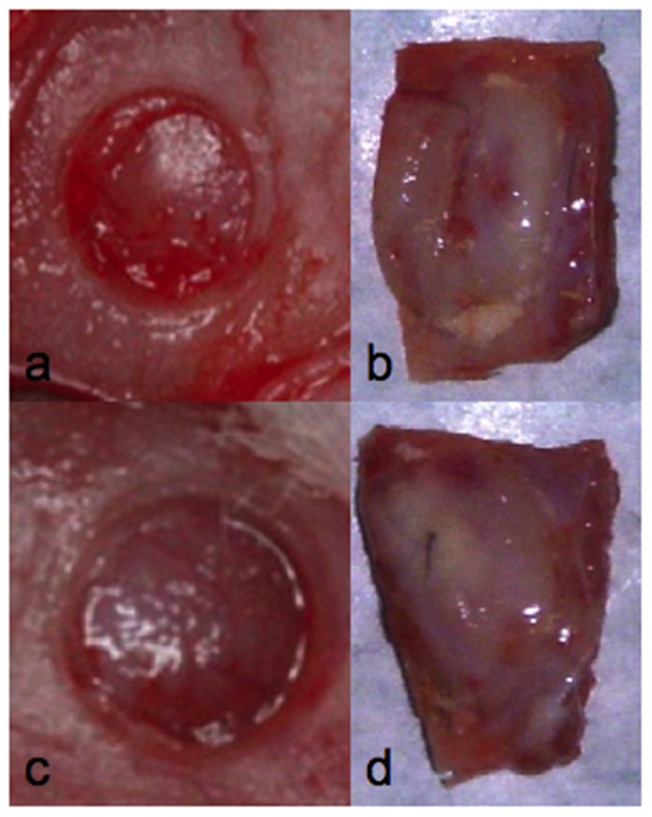
Critical size defect (A, C) before the treatment with DPSCs. Defect after treatment with DPSCs from the younger group (B) and older group (D).

The bone regeneration activity of the DPSCs was assessed in vivo using a rat calvarial defect model (Fig. 8a; 8c). We considered 2 different groups to ascertain the influence of undifferentiated DPSCs on new bone formation: DPSCs from the 16–25-year donor group (Fig. 8 b) and from the over-66 group at p5 (Fig. 8d). As controls, HA granules without cells were used.

**Figure 9:**
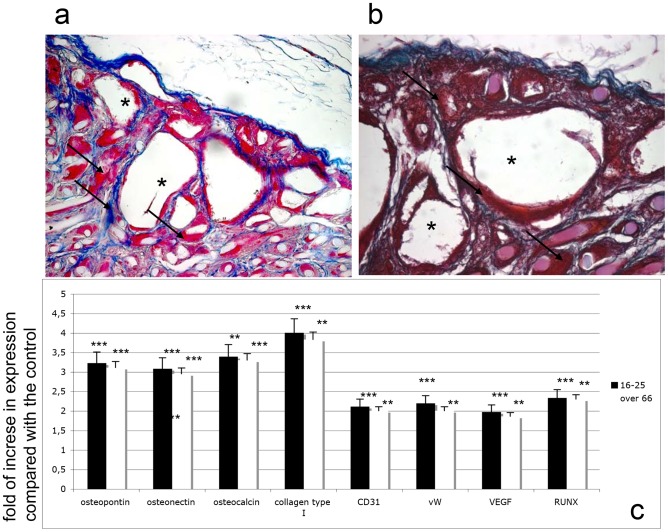
In vivo engraftment of HA nanostructured scaffolds (van Gieson staining, 20×). (A) DPSCs from the younger group (16–25). (B) DPSCs from the senior group (>66). *, HA granules; black arrows, extracellular matrix. (C) Real-time PCR. Time course of osteogenic (osteopontin, osteonectin, osteocalcin, collagen type I, Runx2) and vasculogenic (CD31, vWF, VEGF) mRNA expression analyzed by semi-quantitative real-time PCR of HA nanostructured scaffolds in vivo embedded with DPSCs after 21 days. The results for each experiment are from quadruplicate experiments. Values are expressed as the mean ± SD. T tests were used to determine significant differences (p<0.05). * p<0,05; * * p<0,01; * * * p<0,001.

Concerning the cell populations filling the implants, it was evident that no inflammatory reaction around or inside the implant containing stem cells (Fig. 9) from either donor age group occurred (Fig. 9a from the younger donor group; Fig. 9b from the senior donor group).

In both implants enriched with stem cells, the HA nanostructured granules (Fig. 9c *) were fully embedded with osteoblast-like cells capable of producing a good extracellular matrix consisting mainly of collagen type I, as revealed by van Gieson staining (Fig. 9c, black arrows). A more detailed analysis of the cell population was performed using real-time PCR for osteogenic markers. As shown in Fig. 8c, the presence of HA considerably improved the osteogenic population: the markers for osteopontin, osteonectin, osteocalcin, collagen type I, RUNX, VEGF, CD31, vWF, and vascular endothelial growth factor (VEGF) were more strongly expressed when cells where mixed with HA than in monolayer conditions from both donor age groups.

## Discussion

Stem cells are responsible for the growth, homeostasis and repair of many tissues. These cells could be generally classified as embryonic stem cells (ESCs), which are considered pluripotent thanks to their capacity to give rise to every kind of cell, and adult stem cells, which are less pluripotent and therefore termed multipotent [17], [18]. The best-studied adult stem cells are MSCs, which are available from many adult tissues, such as skin, bone marrow, adipose tissue, umbilical cord blood, and teeth [19], [20]. Adult MSCs are considered a potentially valuable cell-based therapeutic tool for a diverse range of clinical purposes. Indeed, MSCs, in addition to their multipotency, are easy to isolate and culture in vitro and do not carry any ethical issues because of their source [21]. Among the tissues that supply MSCs, the dental field has undoubtedly focused primarily on dental pulp.

There is a population of putative post-natal stem cells or DPSCs that may play a relevant role in reparative dentine formation [22], [23]. DPSCs can be considered a heterogeneous population of MSCs because the dental pulp is composed of both mesenchymal and ectodermic components. Despite the multipotential capabilities of DPSCs, their primary commitment seems to be the production of mineralized tissue, although they can generate functionally active neurons under determined environmental conditions [24].

In the end, as with all MSCs, DPSCs are able to migrate to areas of tissue damage in immune-privileged conditions, and they possess immunosuppressive properties. All these advantages have allowed for successful DPSC transplantations (both autologous and heterologous) [24]–[26].

In light of the above findings, the aim of the present work was the detailed characterization of the stemness properties of DPSCs derived from patients of different ages, so that we could define a simple protocol for the recruitment of a greater quantity of stem cells for clinical application than current protocols provide.

Our first goal was to define the proliferation ability of DPSCs as relates to the age of the patient and to the *in vitro* culture time. In our DPSC cultures, we observed a good proliferative ability for all age classes chosen. This ability was maintained at a high level at p2 in cells from all age groups and during the entire *in vitro* aging process in cells derived from young donors (up to 25 years). A reduction of doubling time was strongly correlated to the increase of the *in vitro* age. This important result for the future of stem cell therapies is supported by the important work of Lizier et al. [31], who found that they could scale up the stem cell production at early passages with minimum risk of losing their stemness. This could be explained by the intriguing results of Mokry et al [30] that correlated the in vitro aging–related doubling time changes to telomere shortening. The authors indeed provided clear evidence of telomere shortening, which we also detected when comparing relative telomere length of DPSCs harvested at different time intervals from culture. It is believed that telomerase activity is responsible for at least partial restoration of telomere DNA strands [15]. Interestingly, the levels of telomerase activity may differ among stem cell populations, which likely reflects their different hierarchical origins [30].

Our second goal was to analyze the commitment ability of DPSCs both qualitatively and quantitatively. DPSCs have well-established stemness properties [11], [15], [16], [22], and thus in our experiments, we quantified the stemness of DPSCs. Immunohistological and gene expression analyses were performed to detect tissue-specific markers for neurons, bone and endothelium. The DPSCs expressed all of the expected specific markers when cultured in the presence of the corresponding differentiation medium. We found positive expression of the endothelial marker CD31, the bone marker osteonectin, the neuronal proteins S100, nestin, and β3 tubulin, and the glial proteins CNPase and GFAP [25], [26]. Similar interesting results were also obtained from Kersis's group [29] on which they studies the in vitro and in vivo differentiation of the DPSCs populations, their developmental potential, immunological compatibility, tissue engineering, and transplantation use in studies in animal models.

Once we established the ability of the cells to undergo lineage commitments, we performed a quantitative analysis of the stemness of the cells from each age class and at each in vitro passage to detect the extent to which the isolated stem cells maintained their commitment ability. DPSCs of all examined classes maintained a high level of differentiation ability. Indeed, for all specific cell markers, we performed a quantitative evaluation (by means of counting positive cells *vs* the total cell number) of the percent of cells committed to a given lineage. For all classes selected (in terms of the age of the patient and in terms of in vitro passages), we found a high rate of correct commitment.

As a final goal, we tested if DPSCs cultured in 3D HA nanostructured scaffolds were able to repair critical size defects and if this performance was donor age related. We choose cells of the most interesting groups: the 16–25-year-old donor group (representing the greatest stemness potential) and the over-66 group (representing the least stemness ability). We tested their osteogenic ability when cultured in monolayers at p5 and compared them to cells cultured in 3D scaffolds and used these cells to repair critical size defects in vitro. The ability of several biomaterials to perform osteoconduction has been studied for many years from several research perspectives with excellent results, such as the work of Lobo et al [32]. Moreover, the improvement of osteogenesis with the addition of stem cells has been observed in animal models, e.g., de Mendonça et al [33], Zheng et al [34], and Gardin et al. [35].

Osteogenic commitment was strongly improved when cells were cultured on 3D nanostructured scaffolds, and interestingly, this ability was comparable between the two donor age groups. When we used nanostructured granules embedded with DPSCs derived from the two age groups to repair critical size defects, we observed that their regenerative abilities were comparable. Extracellular matrix secretion and osteogenic and endothelial markers derived from the two donor age groups were identical.

## Conclusions

Despite some progress, the clinical application of adult stem cells presents many obstacles to formulating safe, simple and reproducible cell-based approaches for tooth repair. It is clear that there is both a clinical need for such treatments and a vast patient resource. Dental stem cells have many advantages, and the results to date suggest that teeth are a viable source of adult mesenchymal stem cells for a wide range of clinical applications. So far, no investigation of the stemness of DPSCs and its correlation with the age of the human donor has been performed.

Our data provide three important insights. The first is related to the possibility of isolating adult stem cells from donors of all ages, including patients up to 67 years old. DPSCs from aged donors show a better proliferative ability at lower in vitro passages (maximum p2). The second important conclusion is that cells isolated from all patients maintain their stemness *in vitro* for a long time. DPSCs seem to have a tendency to differentiate into neuronal and bone cells rather than endothelial cells. This may be correlated to dental pulp histology and physiopathology. The third conclusion is that if DPSCs are cultured on nanostructured HA scaffolds, they can regain their biological properties. Although in monolayer conditions, DPSCs derived from the senior group show a low proliferative ability, leading us to presume that if used for several in vitro passages they would exert a low regenerative ability, when cultured on HA nanostructured granules and used in vivo to repair critical size defects, they show the same ability as the younger group in terms of the time to repair and the quality of extracellular matrix.

We can conclude that DPSCs of all donor ages are undoubtedly good candidates for cell-based therapies in several clinical approaches, especially if combined with nanostructured scaffolds.
